# Philosophical and Psychopathological Perspective of Exile: On Time and Space Experiences

**DOI:** 10.3389/fpsyt.2015.00078

**Published:** 2015-05-27

**Authors:** Matías Silva Rojas, Julio Armijo Nuñez, Gonzalo Nuñez Erices

**Affiliations:** ^1^Université catholique de Louvain, Belgium; ^2^Universidad de Chile, Santiago, Chile; ^3^University of Sheffield, Sheffield, UK

**Keywords:** exile, time experience, space experience

## Abstract

This article addresses the experience of exile from an interdisciplinary perspective (philosophy and psychiatry). The main purpose is to try to understand the experience of exile by rehearsing a psychopathological perspective to address it, so it can help with the treatment of disorders that come with this experience. Furthermore, the article tries to explore the experience and reflection of philosophers and thinkers who, being exiled themselves, tried to understand and explain this radical human experience, focusing on the experience of time and space.

## Introduction

Exile has been something permanent throughout the history of mankind. In ancient times, it may be found the idea that this world dwelled by human beings is not our own home, which has been a mythic vision widespread in various cultural traditions (1). Exile has been viewed as a paradigm in which the notion of human life, at least in western culture, is interpreted as being exiled (2).

The experience of exile, throughout the twentieth century until current times, has became an experience of mass violence and horror, from a political and economical interests of different kinds, rather as only an individual experience, which reclaims us a reinterpretation of the meaning of this experience (3, 4). Actually, in 2013, 10.7 millions of people have been displaced as result of persecutions, political conflicts, generalized violence, or human rights violations, the highest level since global statics about forced displacements are available (5).

The purpose of this paper is to address the theoretical aspects of exile from an interdisciplinary perspective (philosophy and anthropologic psychiatry). This work does not look for highlighting a morbid approach, but moves toward a psychopathological interpretation of the experience of exile vis-à-vis the work of three exiled thinkers who have explicitly thought this topic from a phenomenological view: Eduardo Carrasco (Chile, philosopher) (6), María Zambrano (Spain, philosopher) (7), and José Solanes (Spain, psychiatrist) (1).

It is necessary to clarify that the authors of this paper have not personally experienced any kind of exile. Thus, the main goal of this work has been the attempt to understand an experience, which is unknown vividly to us, through the theorization of exiled thinkers who have tried to explain the meaning and scoops of exile. This raises a methodological difficulty for our work related to the dual status of the texts we will be working on, since they involve both the theoretical and the first-person testimonial. Therefore, since we are theorizing on an experience not lived by any individual, we must move carefully; avoiding any radical schisms at the descriptive level in our search for commonalities among the different authors, and arguments as well as explanations they may provide for these experiences. We welcome this difficulty as a feature without which we could not get the target that we are addressing. Indeed, we believe that in this controversy we can find the contact between theory and experience: the “non-experienced” and the ability to understand it.

### The space and time of exile

The word “exile,” from Latin *exilium*, is derived from *exsilire* (leap out). It is defined as the notion of being forced to leave one’s own space or place (country). The Encyclopedia Britannica highlights the time spent out of that place, which is to be extended. Thus, “exile” refers to those who have been forced to leave their own space for an undetermined and extended time. However, this first definition cannot be easily countersigned: human history offers an endless list of several types of exile, which have been interpreted in different ways. As Solanes points out, difficulties in defining exile and finding the right word for those who live the experience do not involve an abstract concept of time and space, but concrete social and linguistic diversity: “The name of those who live this experience changes according to the language, political or legal point of view regarding the moment in the history” (1). This variety of names shows that none of them explain all what this experience encompasses. Due to the fact that exiled people do not have an agreement about how to name their own condition, the search itself of the meaning about the experience of being exiled shows a feeling of transformation that the exiled person resists suffer passively.

Zambrano addresses exile and the problematic nature of the term polysemy by employing the metaphor of road’s steps: a series of steps from one experience to another that the exiled person walks following their expulsion. According to Zambrano, exile does not feel like such until one begins to feel abandoned (8). This is what specifically differentiates the exile experience from refugees and expatriates. The refugee would be the first step of exile: the refugee finds his space, comfortable or just tolerated, where his body lies after the expulsion. In the second step, the expatriated feel landless (homesick) because he does not feel as own neither the land from which he has been expulsed nor the land in which he has arrived because this new “homeland” cannot replace the original one. Thus, being expatriated does not entail being exiled, but only an expulsion: “the unavoidable distance and the blurred physical presence of the lost country” (8). In this phenomenon, the expulsed person begins to feel close to exile’s border, in its limit, the limit of abandonment. This borderline situation is experienced to the extent that feeling of belongingness has been deprived: “In the abandonment what is already dispossessed appears as own, that which cannot become as belonging to oneself. Belongingness is only negation, impossibility” (8).

Carrasco’s interpretation of exile coincides with Zambrano’s view regarding the non-static condition of this experience. The “leap out” involved in the meaning of “exile” shows that the exile implies the movement of leaving a demarcation; who has carried out that movement is located “outside” of his/her own space or in a different place. According to Carrasco, if the etymology reveals some of the essence of the phenomenon of exile, it is its instability, which can be understood as the condition of being neither in the first nor the second territory: “Exile is to be in any fixed place, not inside or outside, but suspended in the leap; exile is the leap itself from the inside to the outside” (6). How does this suspension of time and space play a role in the life of the exiled person?

As Solanes contends, a significant difference exists between the emotions after the departure into exile and the arrival into the host country. There are many writings, stories, testimonies, or songs about the exile’s departure, but the experiences about the arrival are covered by a blanket of silence. According to Solanes, this is due to these emotions and experiences become so hard to communicate and analyze: “You breathe more deeply when you know you are out of reach of your adversaries; but where is and what is it this place that has provided a safety condition? Surprisingly, one notes that nothing looks like what is known and what was imagined.” (1). Nevertheless, the feeling of facing the unusual or unknown is not due to that the new place is much different to the previous one; it is not about differences between objects, but the relationship that the subject has with the wholeness: the totality of the experienced places. The phenomenon is more an issue of feeling alien rather than differences between places or objects and that feeling is not a problem of quantity: “Alienness is clearly a response to a qualitative diversity that is overall perceived as the fact of discovering that in some dark way – though sometimes just funny, but often disturbing – in exile we are personally related to this larger whole. Being in a qualitatively different world brings the experience of being alien” (1).

The relationship between the exiled with his/her space refers not only to an experience with the host land. This place experienced as alienness is given by the qualitative distance that the exiled person feels about the place left behind. Carrasco interprets the bond between the exiled with that place left backward (his own “country”) as Solanes does. According to the Chilean philosopher, it must be distinguished the original country such as appears in our understanding about it: an abstract disposition of distances, geopolitical organization, our memories of that country, namely: “a world that is dwelled from specific meanings, requirements, obstacles, stimuli, possibilities, objects of desire, fear and different feelings, callings for thought, imagination, strangeness, astonishment, etc.” (6).

Both authors will address this topic from a phenomenological view. While the former states that space or “territory” must be understood “just as it is given in consciousness, namely: such as the phenomenon itself appears as a aroundnesss (vital landscape)” (6), the latter speaks in terms of a “qualitative geography” that emphasizes that any configuration or disposition of space depicts a mode of being, “it is learning, discovery, *invention*” (1).

According to this notion of place or space, we should not understand the idea of country, as Carrasco says, from the current broader sense of Nation, but as the place from which we come from (the homeland), that “original *where*.” Thus, the country or the “original where” would be the place of the “initial disclosedness that determines us as individuals in some specific situation” (6). This means that the country, to the extent that is understood as the “original where” of any traveler, is the reference center of the exiled people’s life who carry that baggage wherever they go. From the phenomenological approach of the intentionality of consciousness assumed by the Chilean philosopher, an individual is constituted by a concrete circumstance. That means that consciousness mirrors itself as a unity with such-and-such landscape. All consciousness is essentially determined or concrete what entails that individuality is a unity between consciousness and the circumstance in which everyone exists. From this perspective, exile can be only shown as a way of living the own belongingness to the country, a way of currying the own country.

Zambrano also recognizes the fundamental meaning of that original space. She argues that the notions of homeland, land, or home refer to the same idea: they are areas or enclosures or different ways of understanding and presenting that “original where” as the lost place; for this reason, the exiled seems to be out of himself in terms of being uprooted because of his lack of home or homeland: “Once we left them, the exiled stays forever outside, exposed to the interpretation of others which look themselves through looking the exiled; something impossible in his own home, his own geography and history: looking to himself in his own roots without dropping them at all, without having been pulled out from him roots” (8). It may be thought that without the limit experience of exile is quite difficult to find out our own “where.” However, that “where” emerges stronger and clearer for the exiled, “like that in which the exile was unconsciously belonging -as Carrasco points out- appears in all its appropriate appearance” (6). Being excluded from returning, the exiled is opened to the concrete possibility of getting the “where” of others, but this is as impossible as it would be a real and effective change of country, in the sense of a change from our “original place” to another one. Exile is then experienced as the impossibility of becoming part of and sharing the “where” of others. At best, exiled people might be able to settle down their own “where,” more or less comfortable, in their host country and thus make it their “adopted country” (6).

Exile appears between two impossibilities: the impossibility of being unable to be in our own homeland, and the impossibility to find a proper replacement for it. This leads to a situation of being “suspended”; that unsteady “betweeness” pointed out by Carrasco’s etymological approach. This is viewed by Zambrano as follows: “The impossibility of living even though you realize the impossibility of dying. The edge between life and death where each one rejects the other. Holding on that limit is the first unavoidable requirement of all exiles” (8). Zambrano develops the idea of the lost of a place and the suspension that involves. According to the Spanish philosopher, the orphanhood is the most important feature of exile: “having no place in the world, neither geographic, social, political nor [.] ontological” (8).

Solanes calls “un-space” (*desespacio*) that place where the exiled dwells, which cannot be objectively located and where distances cannot be estimated by their precise magnitude. Regarding this, Zambrano contends that exile is the space of vastness that comes out gradually. “The vastness, an endless desert, the non-existence of the horizon and the continuous sky, that existent human experimenting that situation has already begun the exile, as an ocean without any island in sight, no real north, destination or goal” (8). It is not about the loss of cardinal points which, as we have seen, represent quantitative differentiates about the “original where,” but it rather refers to the instability of the idea of having reference points. “Even the idea of human space is threatened by this experience in which we do not understand the notion of plan, limits and dispositions; this shows another idea: the limbo’s idea” (1).

Addressing the experience of time represents a somewhat more complex issue. Although each author seems to recognize a similar relevance of the experience of space and time, just Solanes highlights the characteristics that time has in the exiled person’s life. The first thing argued by all of them is that the time’s experience runs parallel with space. Thus, Carrasco contends that to the “original where,” which determines individuality, corresponds an “original time” that is characterized by the individual’s location throughout human history. On the other hand, Solanes talks about “un-time” (*destiempo*) that steams from the idea that the exiled experiences an uprooted space because he/she is depraved of living in the own land; thus, he will undergo the experience of time as “un-time” insofar as he is also expulsed from his original time. Zambrano assumes a similar perspective. She contends that the exiled seems to be located on the periphery of history, which generates the idea that the exiled himself is the past, “a past that is frozen and is only presence” (9). Just like space, the consciousness of time of the exiled must not be understood neither geometrically conception nor quantitatively: “time running in consciousness – as Rivas says – is not comparable with either time of everyday life or that time measured by watches; they artificially divide time into equal or fixed periods” (4).

According to Solanes, there are two affective movements that help us to understand how exiled people experience time; although they may be confused with each other, we still talk about past and future: nostalgia and hope. The nostalgia comes to ensure the continuity of the self and warns against the stranger that appears within the place of the exiled: the exiled not only walks out of his homeland, but also leaves his own self behind him from the exile. Nostalgia, as it were, struggles against the loss of self. “Whoever we have been, we still are (…) nostalgia proclaims personal continuity: suffering warrants it” (1). The hope will appear waiting for the end of the exile, which is thought as finite time; although it is an undetermined moment, there will be an end in the temporality of the experience. Nonetheless, this time of an initial hope will slowly become a wait that becomes longer each day lacking of concrete spaces to holding on (4). The second function of nostalgia in exile would be a future function: living for the return. And this does not mean that the life of exile has no other purpose than to return home; it implies something more radical: that life is pointless only after that the return has taken place.

The exiled will then have a contradictory experience: he notices, on the one hand, just like in the past, time goes on. On the other hand, temporality is tied to the concrete vital space from which the exiled has been expulsed and having no current temporal experience of that. Thus, the exiled will tend to perceive that past time as real space to be reached in the future; personal time will be then measured from the precise time at the moment in which exile had begun. “Will time be resumed on that day? Much like exile will be thought to finish at some point, “un-time” is thought to finish too and will be recovered in the future” (1). The linear conception of time in which past, present, and future are interpreted as continuity becomes obsolete in exile; it seems that is something only possible in the homeland: the originary own space. Therefore, it may be said that in the temporal condition of the exiled, time stands still. Looking at the past with nostalgia and future with hope (1).

### Psychopathological perspective

For irregular migrants settled down in a given territory, expulsion can be a vital disaster (10). In exile, however, return may become a yearning whose essence is the way to experience time and space: lived time and lived space (11). We do not experience temporality in our consciousness as a perpetual succession of different elements. Actually, lived time appears closely related to duration, stability, and flowing phenomena across time. They contain part of time in themselves and constitute temporary figures: the memory with its evocation of the past and the desire and future-oriented hope. It is in these phenomena in which exile can be understood as memory, desire, and hope. Moreover, distance is also interpreted qualitatively; it is not a quantitative distance, but phenomenological. The experience of this phenomenological distance is not linked to displacement or the historical path in exile, but the distance that separates or rather that connects someone with life. Phenomenological distance is completely different from the geometric distance and cannot be overlooked by the exiled because his temporality is always moving with him. This sort of distance connects rather than separates; neither grows nor decreases with being away from objects; and has no boundaries. Thus, while geometric or quantitative distance is affected in exile, qualitative distance does not. Indeed, the latter may be considered as a space to be developed or enhanced; a meeting place with others (11).

Exile in itself is not a pathological experience, but a condition of possibility, a way of being in the world. However, psychopathology manifestations may also occur. A pathological form of experiencing exile in the dimensions of time and space would be in the form of melancholia (melancholic depression). Tellembach proposes a phenomenological vision of what he calls the endogenous-melancholic transformation as a way to develop melancholy in vulnerable people due to their biological (endogenous), psychological (“typusmellancolicus”), and social conditions: “to place means including the aroundness in the existential project. If the other avoids this inclusion, the situation that has been constituted by the same type (“typusmellancolicus”) becomes pathogenic” (12). According to author, the constitution of a vital project ordered in the melancholic is a protection against anxiety to the extent that the anguished requires to live in a safe and known environment, in which randomness does not play any role. From a different epistemology, the idealization of the territory from which the exile has been exiled may also constitute a defense against anguish, which aims to protect the positive aspects of an object – the homeland – from the possibility that negative aspects cover everything completely (13). Tellembach describes certain clinical cases of melancholy linked to specific situations, including melancholy due to uprooting (12).

It should be also noted that exile is a condition that allows for the sociological processes of ethno-cultural exchanges to occur. According to Sonia Montecinos, miscegenation occurs through a process where “the purely biological yields to other processes linked to the history of our territories; the coupling of people is a coupling of cultures: the acculturation that is the mixture of cultural elements, and assimilation – i.e., the absorption of an individual or a people by another culture.” Thus, these two processes will be the axes of the interconnections of miscegenation, which constitute a dynamic that involves simultaneously multiple and intricate biological elements (miscegenation), cultural (moral and symbolic), and socioeconomic (hierarchies and dominations) (14). These processes of acculturation and assimilation will constitute exile cultural exchange, in which the new place becomes a lived space that links rather than separates, where there is room for the exchange of cultures, personal, and human growth.

## Conflict of Interest Statement

The authors declare that the research was conducted in the absence of any commercial or financial relationships that could be construed as a potential conflict of interest.
